# CropPhenoX: high-throughput automatic extraction system for wheat seedling phenotypic traits based on software and hardware collaboration

**DOI:** 10.3389/fpls.2025.1650229

**Published:** 2025-08-07

**Authors:** Jinxing Wang, Baohua Yang, Pengfei Wang, Runchao Chen, Hongbo Zhi, Zhiyuan Duan

**Affiliations:** School of Information and Artificial Intelligence, Anhui Agricultural University, Hefei, China

**Keywords:** CropPhenoX, phenotypic trait, software-hardware collaboration, wheat, highthroughput detection

## Abstract

Accurately quantifying wheat seedling phenotypic traits is crucial for genetic breeding and the development of smart agriculture. However, existing phenotypic extraction methods are difficult to meet the needs of high-throughput and high-precision detection in complex scenarios. To this end, this paper proposes a high-throughput automated extraction system for wheat seedling phenotypic traits based on software and hardware collaboration, CropPhenoX. In terms of hardware, an architecture integrating Siemens programmable logic controller (PLC) modules is constructed to realize intelligent scheduling of crop transportation. The stability and efficiency of data acquisition are guaranteed by coordinating and controlling lighting equipment, cameras, and photoelectric switches. Modbus transmission control protocol (TCP) is used to achieve real-time data interaction and remote monitoring. In terms of software, the Wheat-RYNet model for wheat seedling detection is proposed, which combines the detection efficiency of YOLOv5, the lightweight architecture of MobileOne, and the efficient channel attention mechanism (ECA). By designing an adaptive rotation frame detection method, the challenges brought by leaf overlap and tilt are effectively overcome. In addition, a phenotypic trait extraction platform is developed to collect high-definition images in real time. The Wheat-RYNet model was used to extract wheat seedling phenotypic traits, such as leaf length, leaf width, leaf area, plant height, leaf inclination, etc. Compared with the actual measured values, the average fitting determination coefficient reached 0.9. The test results show that CropPhenoX provides an intelligent integrated solution for crop phenotyping research, breeding analysis and field management.

## 1 Introduction

The phenotypic traits of wheat seedlings directly reflect their growth status, physiological characteristics, and genetic potential (Crain et al., 2017). Accurate acquisition of these phenotypic traits is crucial for predicting wheat yield, evaluating quality, and formulating field management strategies (Pour-Aboughadareh et al., 2020; Akram et al., 2008). However, traditional methods are insufficient in terms of efficiency and automation to meet the needs of accelerating breeding, transforming production methods, and ensuring food security. Therefore, it is necessary to develop an automated phenotypic trait monitoring platform (Zhang et al., 2024), which is of great significance for shortening the breeding cycle and accelerating genetic improvement.

Traditional methods for obtaining crop phenotypic parameters often rely on manual measurement. They have problems like strong subjective interference, low efficiency and poor real-time performance. These greatly limit the efficiency of large-scale genetic breeding screening (Mohanty et al., 2016). Studies have shown that crop phenotypic detection methods based on three-dimensional information can better reflect the morphological and structural characteristics of crops. Shlyakhter et al. (2001) used the L-system to simulate tree growth and used stereo matching and calibration of binocular vision. Andújar et al. (2016) used the Kinect v2 depth camera to collect lettuce images from multiple angles for three-dimensional reconstruction. Yang et al. (2017) used the TOF (Time-of-Flight) depth camera to collect serial point cloud images of red peppers to achieve automatic extraction of phenotypic parameters. The above research shows that 3D models can accurately describe the spatial morphology of crops. However, high equipment costs, time costs, and complex algorithms often limit their widespread promotion. Visible light imaging, with its low cost and high efficiency, is better suited for measuring morphological parameters (Li et al., 2014).

Recently, computer vision and deep learning have advanced rapidly. This has brought unprecedented opportunities for automated phenotyping, breaking traditional limitations significantly (Koh et al., 2021). These technologies efficiently process large-scale image data and extract diverse phenotypic features. Notably, convolutional neural networks, with strong nonlinear modeling and complex data pattern mining capabilities, enable automated extraction of crop phenotypic parameters (Xiong et al., 2021). Previous studies have shown that convolutional neural network (CNN) are widely used in image-based plant phenotyping analysis (Arya et al., 2022). Lee et al. (2017) also used CNN to successfully train a model for effectively distinguishing 44 plant leaves. Deep learning-based wheat ear detection, such as fully convolutional networks (FCN) (Wang et al., 2019), Faster-RCNN (Madec et al., 2019), and dual-stage target detection network models, such as EfficientDet-D0 (Wang et al., 2021), YOLO v3 (Yang et al., 2019b), YOLO v4 (Yang et al., 2021), and YOLO v5 (Dandrifosse et al., 2022; Zang et al., 2022), can all achieve accurate wheat ear detection. Deep learning enables wheat phenotypic parameter acquisition. But existing studies use horizontal bounding boxes for wheat ear detection, failing high-throughput needs. These boxes lack angle regression and target directionality, hindering accurate extraction of wheat seedling phenotypes (Nie and Huang, 2022). Moreover, during training and inference with horizontal boxes, candidate targets generate numerous redundant bounding boxes, which easily incorrect detection and missed detection of wheat leaves, leading to inaccurate positioning (Liu et al, 2022). Therefore, wheat leaf positioning and detection, as a prerequisite for phenotypic parameter extraction, still face many challenges.

In fact, rotating target detection can obtain the precise position and direction information of the target by detecting the target with a rectangle with a rotation angle (Chen et al., 2024), which provides a new path to improve the accuracy and robustness of wheat leaf detection. Sun et al. (2022) proposed an improved rotating bounding box wheat head detection and counting model to detect and count wheat ears in the field environment from a bird’s-eye view. The research shows that rotating bounding box detection is helpful for extracting wheat phenotypic parameters. In addition, there are other rotated bounding box detection models, such as Roi trans (Ding et al., 2018), SCRDet (Zhao et al., 2023), and R-YOLOv5 (Li et al., 2022), which have been paid attention to and applied by many scholars. However, the effect of wheat seedling detection based on these detection models with rotated bounding box is still unknown. In particular, the detection and phenotypic parameter extraction of wheat seedlings face the following problems. On the one hand, the leaves of wheat seedlings are small, with different shapes and arbitrary growth directions, making it difficult for traditional detection methods based on horizontal bounding boxes (HBBs) to accurately capture the complete outline and posture of the leaves. On the other hand, the shape of wheat leaves is narrow and has a large aspect ratio, which causes typical horizontal detectors to miss detection, thereby affecting the extraction accuracy of key phenotypic parameters such as leaf area, length, and width (Li et al., 2024; Chen et al., 2024). Therefore, it is necessary to design an efficient rotating bounding box detection model to realize the automatic and accurate identification and positioning of wheat leaves in the seedling stage, which has become a current technical challenge.

To this end, the Wheat-RYNet, a detection model for wheat leaves based on rotated bounding boxes, is proposed in this study. Meanwhile, an automated analysis platform and a high-throughput phenotyping system are developed to achieve accurate detection of wheat leaves and efficient extraction of phenotypic traits. The specific contributions are as follows:

1. The Wheat-RYNet, a detection model for wheat seedlings based on rotated detection boxes, is proposed. By utilizing the precise information of the rotated bounding boxes, it is able to accurately calculate the core traits of wheat seedlings, such as leaf area, leaf length, and leaf width.
2. An automated platform for wheat image acquisition and phenotypic traits extraction has been constructed. The platform incorporates the rotation bounding box detection and trait extraction algorithms, features a user-friendly interface, and enables batch processing of images as well as visualization of the results.
3. A high - throughput crop phenotyping system has been developed, which incorporates high-resolution imaging and intelligent control modules. Through non-contact scanning and automated assembly lines, it enables quick capture of images of wheat seedlings. Combined with detection models and extraction algorithms, it efficiently outputs phenotypic data, providing hardware support for breeding and monitoring.

## 2 Hardware system and software platform

### 2.1 CropPhenoX: high-throughput crop phenotyping system

#### 2.1.1 Hardware components of high-throughput crop phenotyping system

**Figure 1:**
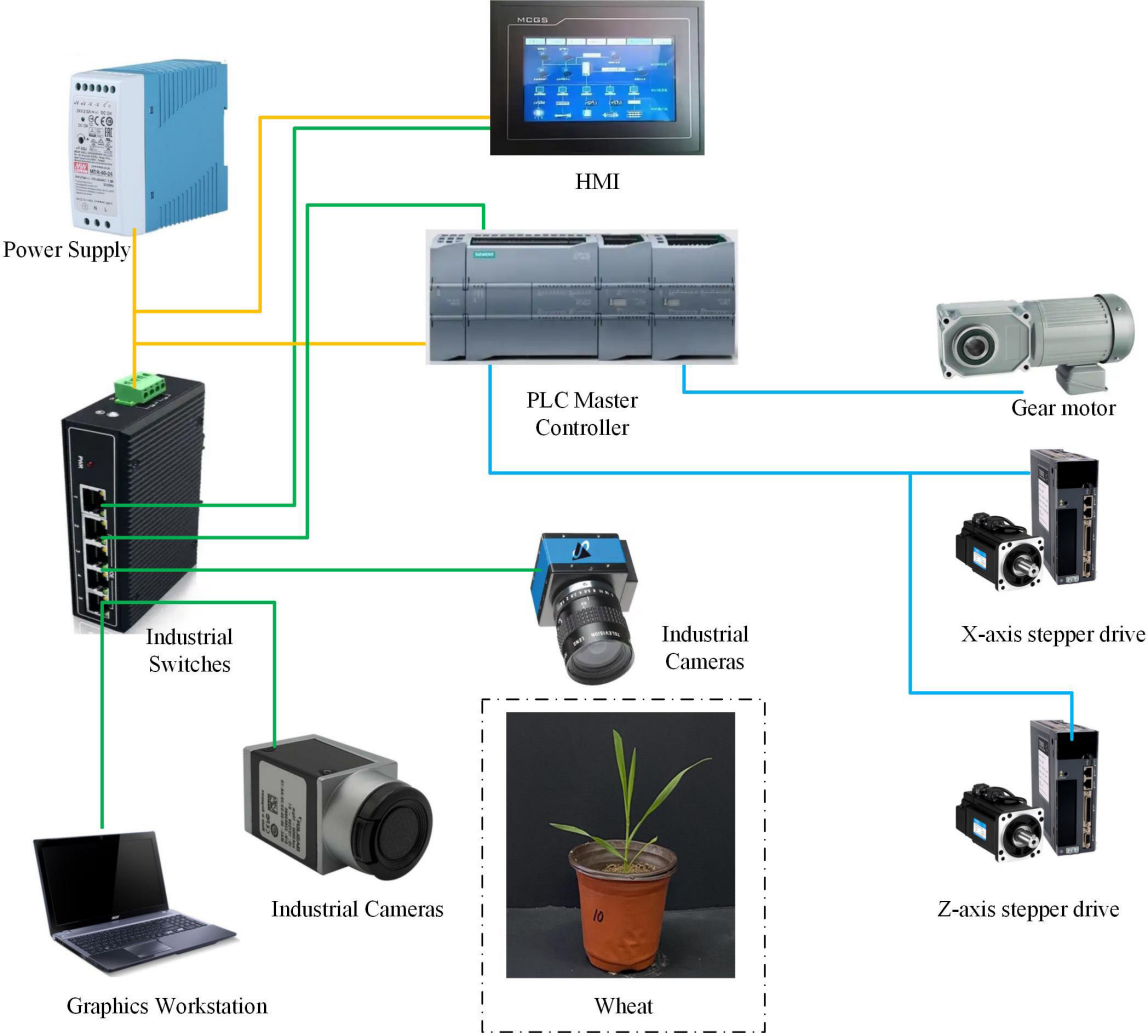
Electrical diagram of high-throughput crop phenotyping system.

To further realize the automatic collection and intelligent analysis of phenotypic information of wheat seedlings, we developed a high-throughput crop phenotyping system, CropPhenoX, as shown in **Figure 1**. The system is built on the Siemens SIMATIC S7–200 SMART series PLC (Programmable Logic Controller) module, which is designed for automated crop transmission track control and crop data collection processes, and has high integration and flexibility. In terms of automated crop transmission track control, the PLC module uses logic programming to achieve precise scheduling of the transmission track, ensuring that potted crops can be automatically transported to the designated workstation according to the preset path, and supports dynamic path planning to avoid conflicts when multiple workstations work together.

In the process of crop data collection, the PLC module integrates the linkage control function of bar light sources, industrial cameras and photoelectric switches. Through high-speed pulse output technology, the system can synchronously control camera exposure and light source brightness, and combine the precise triggering of photoelectric switches to build a stable lighting and triggering environment in the darkroom, so as to efficiently collect key traits such as crop morphology and color.

To meet the needs of data transmission and remote monitoring, the PLC module uses the Modbus TCP protocol as the communication interface to establish a real-time data link with the workstation. The system can upload the collected data and equipment status information to the monitoring platform, and supports multi-level data processing and analysis functions. The workstation can perform real-time analysis, storage and visualization of the transmitted data.

In terms of flexible control, the system dynamically adapts the flexible plate chain and the camera moving module through the PLC module. This design significantly improves the scalability and adaptability of the system, and provides solutions for the diversified scenarios of agricultural production.

#### 2.1.2 Software platform for extracting crop phenotypic traits

**Figure 2:**
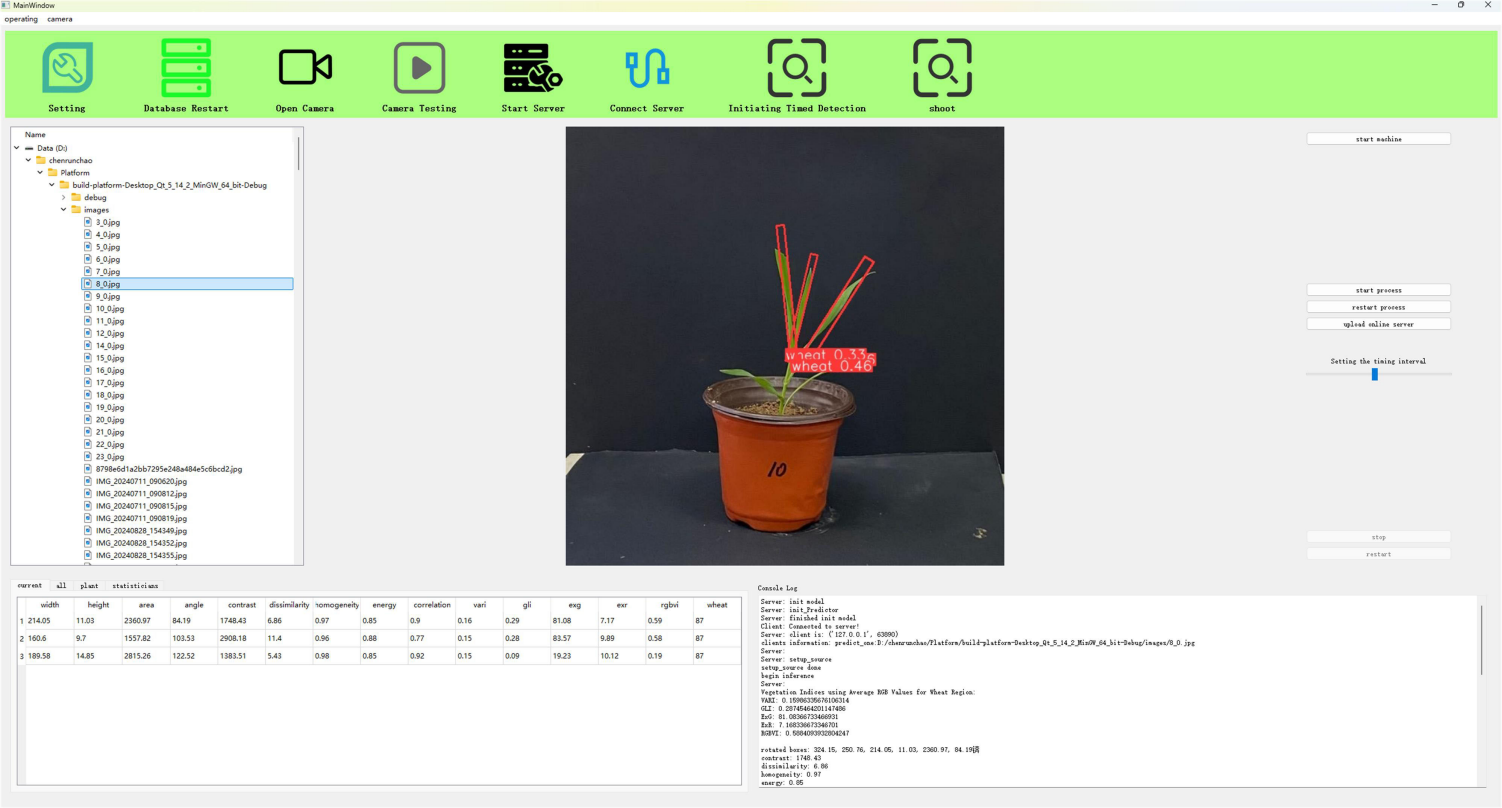
Phenotypic traits extraction platform.

To achieve batch acquisition of wheat phenotypic traits, we developed a phenotypic extraction system, as shown in **Figure 2**. This software system is implemented using Qt Creator 4.11.1, the QT framework uses version 5.14.2, and the C++ compiler uses MinGW 64-bit. The system mainly extracts crop phenotypic information using a rotating target detection model, and mainly includes a toolbar, an operation bar, a file directory management area, an image display area, a hardware operation area, a result display area, and a console.

Among them, the toolbar is used for some basic operations and parameter settings. The operation bar is used for basic operations required by the platform. The file directory management area is used to display the basic distribution of file directories. The image display area is used for image preview, display of shooting, and detection results. The hardware operation area mainly controls the basic operations of the device, and the result display area is used to display plant phenotypic detection results, crop storage conditions, and statistical information. The console is used to output console information during the operation of the platform.

The plant phenotype extraction system based on the rotating object detection model has a user-friendly and concise interface. The system can obtain real-time images of crops and extract crop phenotypic traits based on a certain hardware platform, such as industrial cameras and other image acquisition devices, and display the parameters in real time.

#### 2.1.3 Hardware and software collaboration

**Figure 3:**
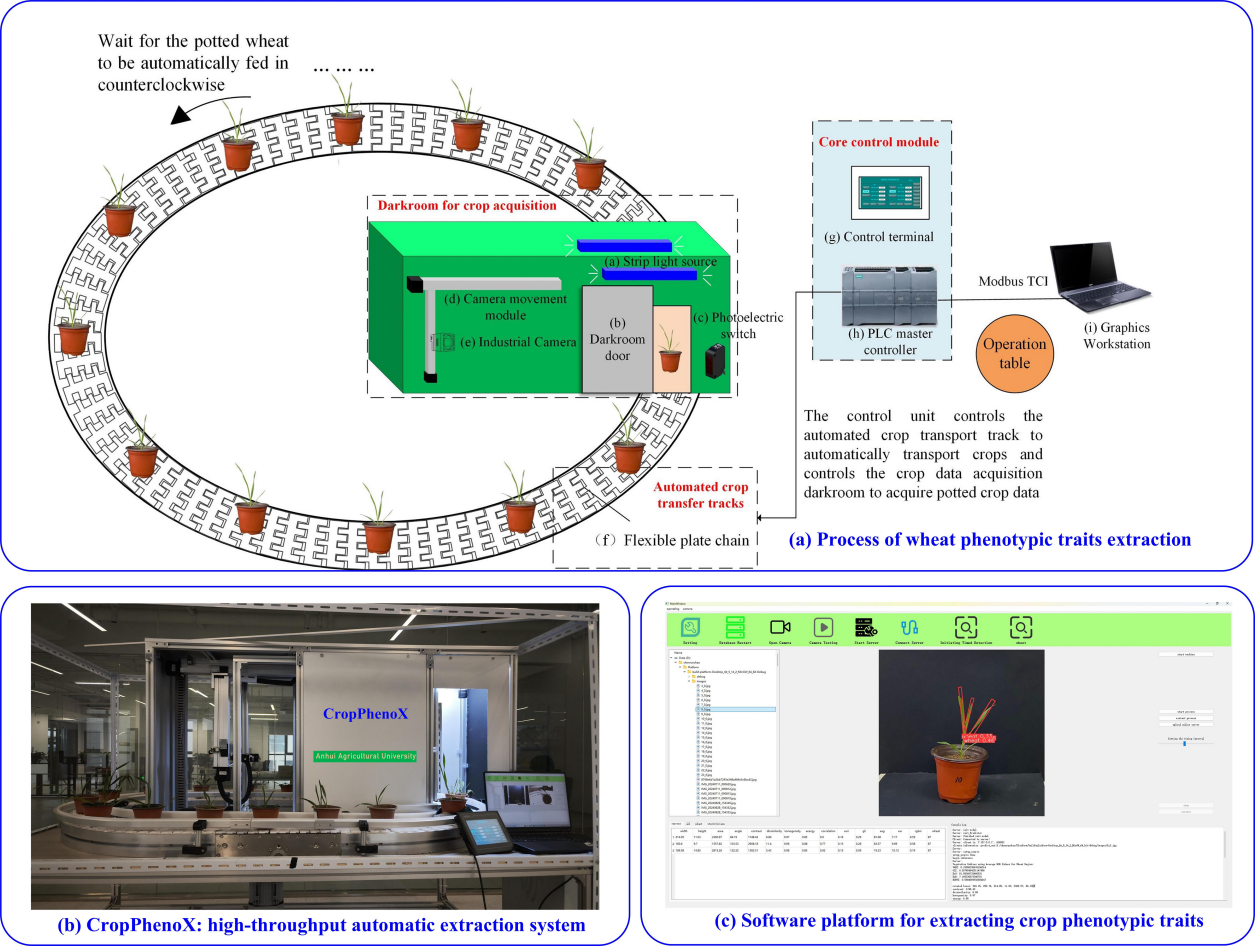
CropPhenoX: High-throughput crop phenotyping system: **(a)** Process of wheat phenotypic traits extraction, **(b)** CropPhenoX: high-throughput automatic extraction system, **(c)** Software platform for extracting crop phenotypic traits.

In the “CropPhenoX” system, the deep integration of hardware and software is the core of realizing the automated and accurate detection of wheat phenotypes. As shown in **Figure 3**, through the coordinated operation of hardware and software platforms, data collection, transmission, and analysis and processing are realized.

In particular, when multiple pots of wheat seedlings are transported to the dark box along the ring chain, the camera automatically captures images, which are uploaded to the server through the wheat phenotyping system and saved in the specified path (**Figure 3b**). The Phenotype extraction system not only displays these images in real time, but also uses our innovative deep learning model to automatically extract the phenotypic traits of the wheat seedlings. On the one hand, the phenotypic traits acquisition device (**Figure 3a**) integrates a mechanical structure, power management system, motion control module, human-computer interaction interface and sensor system. The core is the image acquisition part. To collect images of wheat, an industrial camera (MV-HP120GCe, Weishi, Xi’an, China) is installed on the lifting platform in the dark box. The industrial camera has a built-in advanced metal oxide semiconductor (CMOS, Weishi, Xi’an, China) photosensitive element and a lens model BT-23C0814MP5 (8 mm focal length) to ensure image clarity and detail capture. The camera has a resolution of up to 4508 × 4096 pixels, providing a solid foundation for accurate analysis of crop phenotypic characteristics. The camera is installed on the front liftable platform, keeping it horizontally facing the ground, capturing crop images at an optimal shooting distance of 0.5 meters to ensure data accuracy and consistency.

On the other hand, to further improve user experience and data processing efficiency, we independently developed a crop phenotypic traits extraction system (**Figure 3c**). The system not only supports real-time image preview, allowing users to instantly adjust imaging parameters to obtain the best image quality, but also realizes automated shooting control, flexible parameter configuration, and convenient server startup functions. The integration of this series of functions greatly simplifies the operation process and accelerates the entire process from image acquisition to phenotypic traits extraction, providing strong technical support for research and practice in crop genetic improvement, precision agricultural management, etc.

### 2.2 Data acquisition, labeling, and augmentation

#### 2.2.1 Collection of wheat images

Wheat phenotypic data collection relies on the hardware system of the self-developed CropPhenoX platform. Considering the compact size and moderate height of wheat plants, the camera installation position does not require frequent adjustments. In the initial zero state, the camera is placed at a horizontal distance of 950 mm and a vertical height of 180 mm from the wheat plant to ensure that the imaging field of view fully covers the crop. During data collection, wheat is transported to the darkroom detection area at a uniform speed via a flexible plate - chain conveyor, which triggers the industrial camera to synchronously capture image data. To ensure image quality, the system is equipped with a strip light source for uniform fill lighting, and the image gain parameter is set to 5.0, effectively enhancing image contrast and detail clarity. Meanwhile, to achieve efficient and stable data transmission, the data transmission packet size is configured as 2400 bytes to prevent data packet loss and transmission delays. The flexible plate - chain conveyor continues to operate in a loop until the data collection task for the entire batch of wheat samples is completed, ensuring the integrity and consistency of data acquisition.

The wheat samples used in this study include “Annong 0711” and “Annong 1589”. Annong 0711 is of the upright type, with a compact plant type at the seedling stage;, and Annong 1589 is of the semi-upright type, with a relatively loose plant type at the seedling stage. To systematically improve the diversity of experimental samples, a multi-dimensional acquisition strategy was adopted: on the one hand, images of single, double, triple and quadruple wheat plants were collected; on the other hand, the same plant was photographed from multiple angles, and a total of 269 original images were obtained. After strict manual screening and quality assessment, a total of 244 high-quality wheat images were retained. All images were standardized to a resolution of 3072×3072 pixels and stored in.jpg format to ensure data consistency and availability.

#### 2.2.2 Data annotation with rotated bounding box

**Figure 4:**
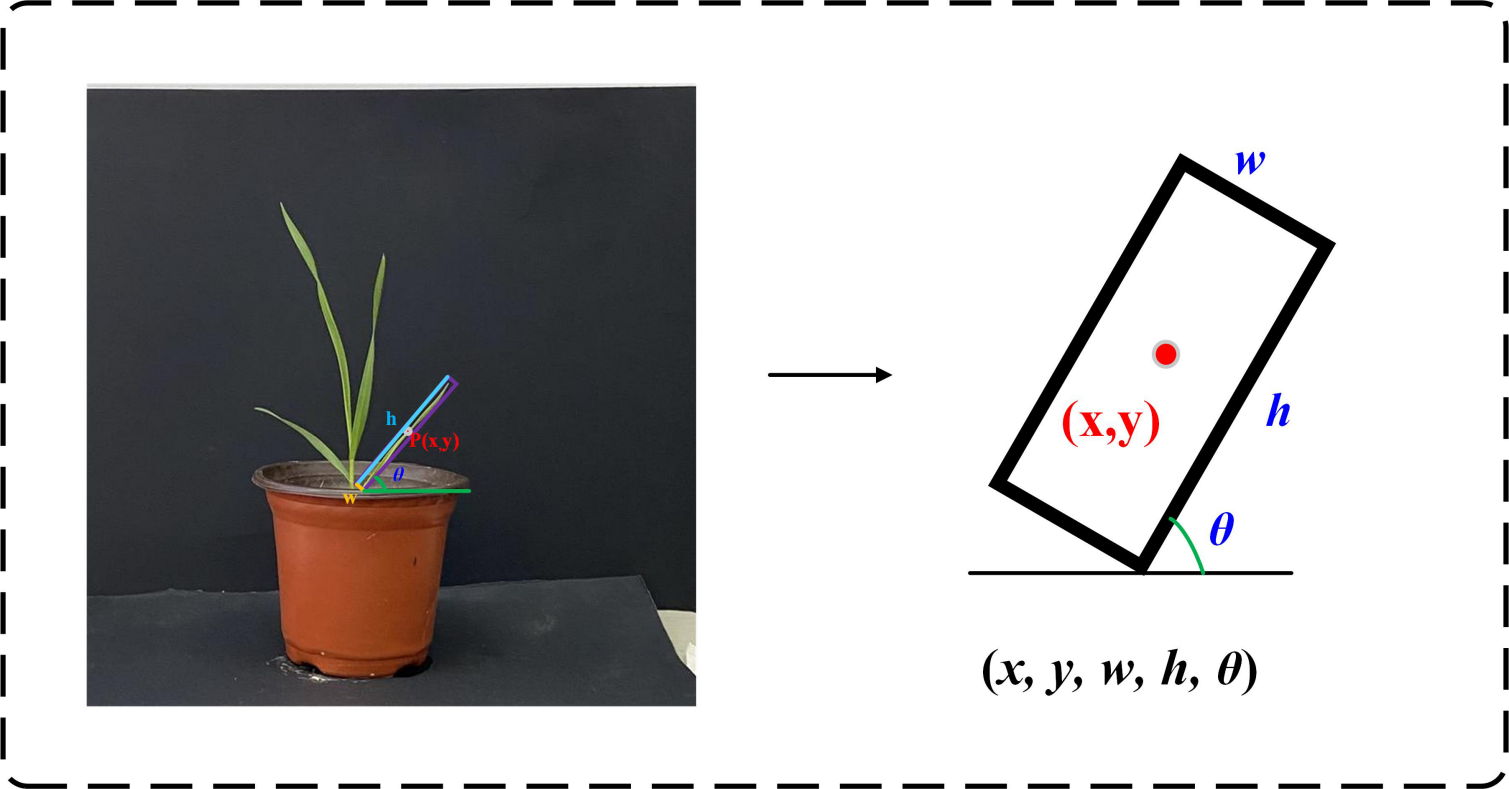
Five parameters annotation method. Where (*x, y*) is the coordinate of the center point, *h* is the length of the long side, *w* is the length of the short side, and *θ* is the counterclockwise rotation angle around the center point.

In the rotating box positioning technology, the more accurate the label annotation method, the less redundant information is provided to the network training. To accurately capture the unique properties of the rotating wheat leaf target, the label not only covers the category of the wheat leaf target and the coordinate information of the axis-aligned bounding box, but also specifically includes the crucial parameter of the rotation angle. As one of the annotation methods, the long side definition method uses five parameters (x,y,w,h,θ) to accurately describe the rotating box, as shown in **Figure 4**. Among them, (x, y)​ is the coordinate of the center point, w is the horizontal width, h is the vertical height, and σ is the counterclockwise rotation angle around the center point ( θ ∈ [−90, 90]).

To improve detection efficiency, the original wheat image was uniformly cropped to 640×640 pixels. Due to the narrow shape and dense distribution of wheat leaves, the use of rotation box annotation can more accurately represent the wheat ear area and avoid more background in the box. In this study, the roLabelImg (https://github.com/cgvict/roLabelImg) annotation tool was used to annotate the area where each wheat ear leaf in the original image was located with a rotation box.

**Figure 5:**
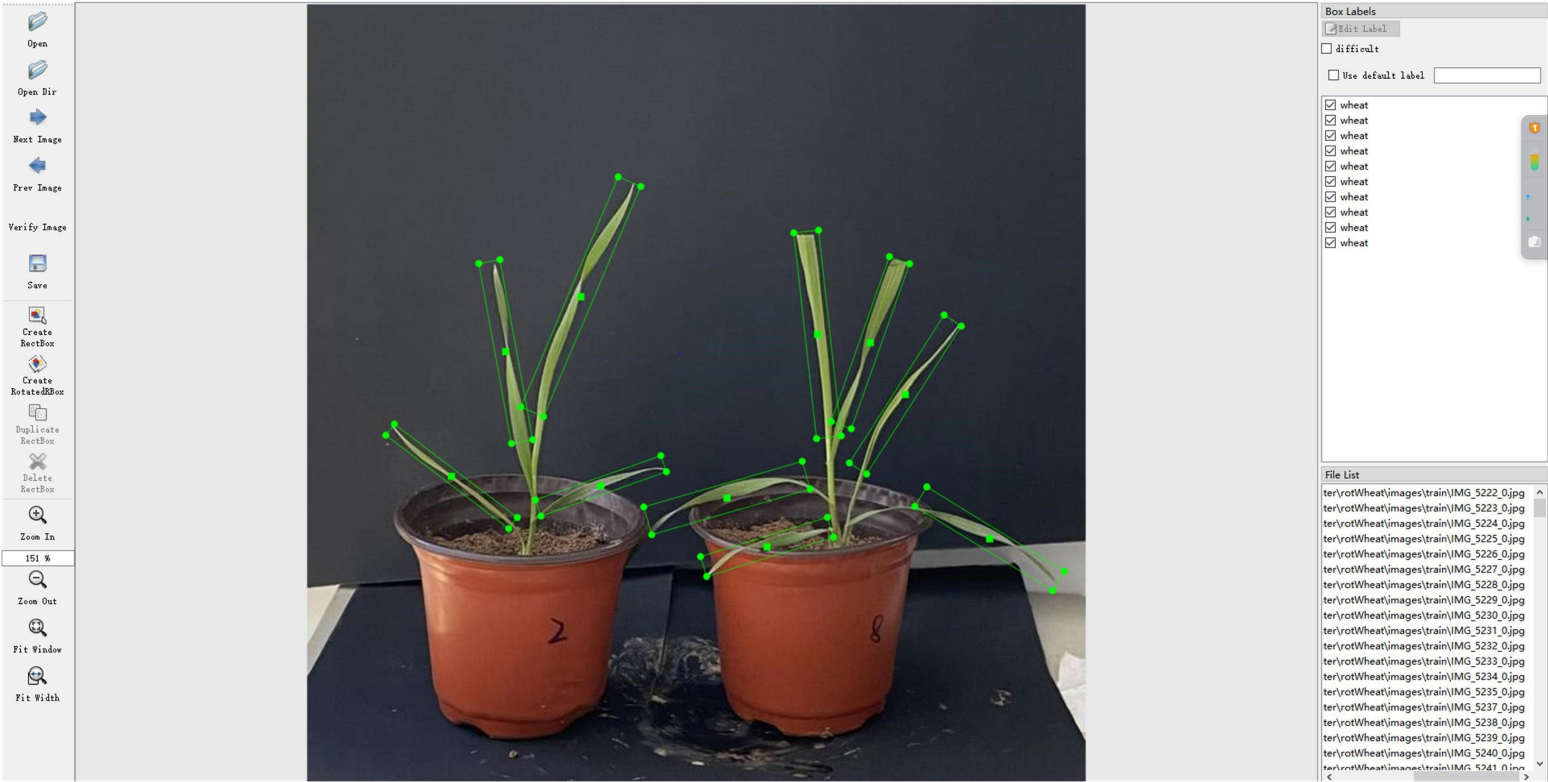
Annotation of wheat seedling images.

The annotation file (.xml) was generated in the PASCAL VOC format. The information of the rotation box annotation is represented as (x,y,w,h,θ) , which respectively represents the coordinates of the center point of the wheat leaf bounding box (x,y) , the length of the short side and the long side (w,h) , and the rotation angle θ compared to the horizontal box. An example of wheat annotation is shown in **Figure 5**. Due to the need of model training, the PASCAL VOC annotation file (.xml) is converted into a DOTA annotation file (.txt). The position of the rotation box is represented by eight parameters, namely the four vertex coordinates $\left(x_{1},y_{1}),(x_{2},y_{2}),(x_{3},y_{3}),(x_{4},y_{4}\right)$ .

#### 2.2.3 Data argument

To enhance the diversity and richness of the data, we adopted a variety of strategies. Firstly, starting from the perspective of image capture, we obtained multi-view images of wheat leaves by rotating them at different angles (such as 45°, 90°, 135°, and 180°). Secondly, we applied a series of image preprocessing methods, including image flipping, mirroring, brightness adjustment, image denoising, and random cropping, to further increase the variability of the samples. Through these methods, we expanded the original 244 wheat image data to 1220. These methods not only help to improve the generalization ability of the model, but also alleviate the challenges brought by data imbalance to a certain extent, ensuring that the model can fully learn the characteristics of wheat leaves during training. The augmentation wheat data set was divided into training set, test set, and validation set in a ratio of 7:2:1, resulting in 854 training sets, 244 test sets, and 122 validation sets.

### 2.3 Wheat seedling detection model

#### 2.3.1 MobileOne

MobileOne is a lightweight network architecture built on the foundation of MobileNet (Vasu et al., 2023). It inherits the efficient depthwise separable convolution module of MobileNet to simplify network parameters and computation, and uniquely introduces the concept of overparameterization to enhance the network’s expressiveness and adaptability.

In MobileOne, the depthwise separable module of MobileNet is replaced by a reparameterized depthwise separable module. Specifically, the depthwise convolution module of MobileOne consists of three parallel processing paths. The first is an overparameterized 3×3 convolution path, which contains *k* reparameterized 3×3 convolution kernels, where *k* is the number of input channels. The second path is a 1×1 convolution path, and the third path is a normalized skip - connection path. This is the architecture of the depthwise convolution module during the training phase. During the testing phase, the reparameterized structure is used for prediction.

The pointwise convolution module of MobileOne consists of two parallel paths. One is an overparameterized 1×1 convolution path, where the number of convolution kernels is determined by the number of input channels. The other is a standardized skip connection path. During testing, the reparameterized structure is also used for prediction.

#### 2.3.2 ECA attention module

The ECA module first performs global average pooling on the input feature map, aiming to extract the global feature vector of each channel (Wang et al., 2020). For the input feature map $X\in R^{B\times C\times H\times W}$ , where *B* is the batch size, *C* is the number of channels, *H* and *W* are the height and width of the feature map, respectively, the calculation formula is shown in Equation 1, where ${\text{z}}_{\text{c}}$ is the pooling value of the c-*th* channel.


(1) $${\text{z}}_{\text{c}}=\frac{1}{H\times W}\sum_{\text{i}=1}^{H}\sum_{\text{j}=1}^{W}X_{c,i,j}$$

The ECA module extracts the global feature vector through global average pooling, and then adaptively selects an odd-sized one-dimensional convolution operation to perform information interaction along the channel dimension to share weights and generate a weight vector. Subsequently, the weight vector is converted into an attention weight using the Sigmoid activation function to achieve dynamic adjustment of the importance of each channel feature. Finally, the attention weight is multiplied by the original input feature map channel by channel to obtain a weighted output feature map, which enhances the network’s sensitivity to key wheat leaf features. The ECA mechanism has become an indispensable attention module in modern convolutional neural networks with its efficient computing and significant feature enhancement capabilities (Xue et al., 2021).

#### 2.3.3 Improved target frame positioning method

In rotated bounding box detection, the design of the localization loss needs to take into account the periodicity of the rotation angle. The loss function $L_{\text{CIoU}}$ of detection model mainly consists of three parts: classification loss ( $L_{\text{class}}$ ), confidence loss ( $L_{\text{conf}}$ ) and positioning loss ( $L_{\text{loc}}$ ). The details are shown in Equations 2-4.


(2) $$L_{\text{CIoU}}=L_{\text{class}}+L_{\text{conf}}+L_{\text{loc}}$$

The classification loss uses binary cross entropy loss, and the specific loss function formula is:


(3) $$y_{i}=\text{Sigmoid}(x_{i})=\frac{1}{1+e^{-x_{i}}}$$

(4) $$L_{\text{class}}=\sum_{n=1}^{N_{class}}{\text{y}}_{i}^{*}\text{log}(y_{i})+(1-{\text{y}}_{i}^{*})\text{log}(1-y_{i})$$

Where: Sigmoid is the name of the activation function; $N_{class}$ is the total number of categories; $x_{i}$ is the predicted value of the current category; $y_{i}$ is the probability of the current category after the activation function; $y_{i}^{*}$ is the true value of the current category (0 or 1); $L_{class}$ is the classification loss.

The confidence loss also uses cross entropy loss, as shown in Equation 5.


(5) $$L_{\text{conf}}=\sum_{i=0}^{s^{2}}\sum_{j=0}^{B}\beta_{ij}^{obj}[{\left(C_{i}-\hat{\text{C}}\right)}^{2}]+\lambda_{noobj}\sum_{i=0}^{s^{2}}\sum_{j=0}^{B}\beta_{ij}^{obj}[{\left(C_{i}-\hat{\text{C}}\right)}^{2}]\;$$

Where: $S^{2}$ represents the prediction box; $C_{i}$ is the confidence score; $\hat{C}$ is the intersection of the prediction box and the true box. When there is an object in a cell, $\beta_{ij}^{obj}$ takes 1, otherwise takes 0, that is, $\beta_{ij}^{noobj}$ takes 0; $\lambda_{noobj}$ represents the weight coefficient, which can reduce the weight of the object-free loss calculation part.

The positioning loss function is as follows:


(6) $$L_{\text{loc}}=\frac{\lambda_{1}}{N}\sum_{n=1}^{N}obj_{n}\sum_{j\in \{x,y,w,h,\theta_{reg}\}}L_{reg}(v_{nj}^{'},v_{nj})+\frac{\lambda_{2}}{N}\sum_{n=1}^{N_{anchor}}L_{\text{CSL}}(\theta_{n}^{'},\theta_{n})+\frac{\lambda_{3}}{N}\sum_{n=1}^{N}L_{\text{class}}(p_{n},t_{n})$$

Where: $N_{anchor}$ is the number of anchor points; $obj_{n}$ is a binary value, which takes 1 for positive samples and 0 for negative samples; $v_{nj}$ is the target true value; $v_{nj}^{'}$ is the predicted offset; $\theta_{n}$ is the angle of the annotation box; $\theta_{n}^{'}$ is the angle of the prediction box; $t_{n}$ is the type label of the identified object; $p_{n}$ is the distribution of the identified object label value calculated by the Sigmoid function; $\lambda_{1}$ , $\lambda_{2}$ , $\lambda_{3}$ are hyperparameters, where $\lambda_{1}$ =1, $\lambda_{2}$ = 0.5, $\lambda_{3}$ =1; $L_{reg}$ represents the regression loss; $L_{\text{CSL}}$ represents the cross entropy loss calculated by the Sigmoid function.

#### 2.3.4 Wheat-RYNet model

**Figure 6:**
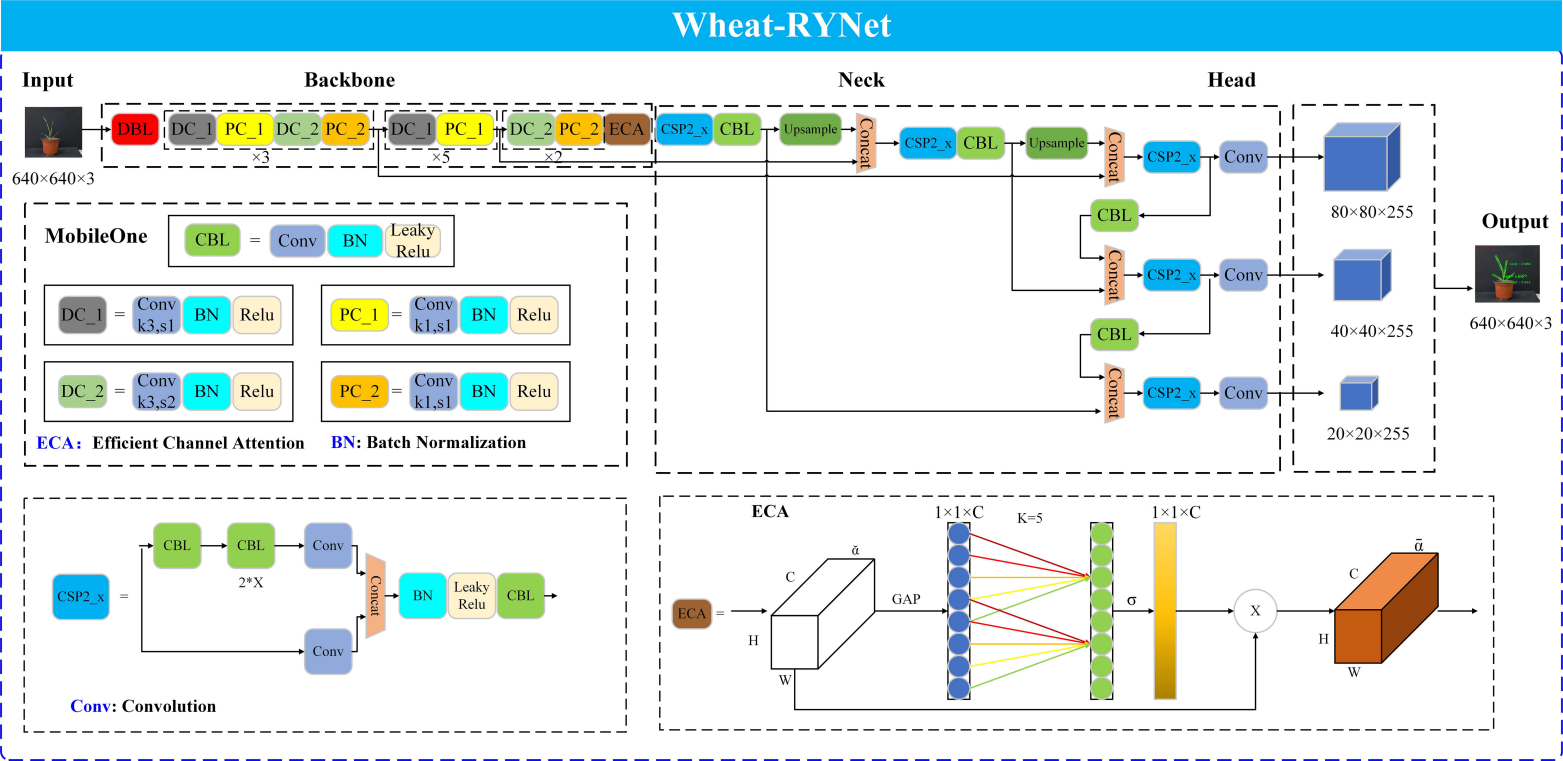
Structure of Wheat-RYNet.

Wheat leaf detection is an important prerequisite for obtaining wheat seedling phenotypic trait. To improve the accuracy of wheat seedling phenotypic traits extraction, the Wheat-RYNet model is proposed to enhance its performance in wheat leaf detection and thus improve the accuracy of phenotypic traits extraction. The structure of the model is shown in **Figure 6**.

Firstly, the backbone network of the model is replaced. YOLO v5 is selected as the basic model, and the original backbone network is replaced with the MobileOne network, which uses deep separable convolution technology to decompose traditional convolution into deep convolution and point-by-point convolution, effectively reducing the number of parameters and reducing the computational complexity, thereby ensuring high accuracy of wheat leaf detection.

Secondly, in the neck component of the Wheat-RYNet architecture, specifically after the CSP2_x module and before the final convolutional layer of the detection head, the network incorporates the ECA (Efficient Channel Attention) attention mechanism. The ECA module avoids the use of global pooling and fully connected layers by directly interacting with features locally in the channel dimension, thereby reducing the computational cost. At the same time, the ECA module can intelligently analyze the importance of each channel feature, give higher weights to key wheat leaf features, weaken the influence of irrelevant features, and improve the network’s sensitivity to target features. In addition, the ECA module structure is simpler, achieving dual optimization of model complexity and attention learning efficiency.

As the core component of the Wheat-RYNet model, the output is set to adapt to specific detection tasks. In the study, for the directionality and morphological diversity of wheat and seedling leaves, the output of the detection not only includes the category confidence of the leaves and the coordinates of the axis-aligned bounding box, but also specifically introduces the rotation angle parameter, so that the model can more accurately capture the position, direction and morphology of the leaves, effectively reducing the interference of background information, thereby improving the accuracy and reliability of wheat leaf target positioning.

Finally, the loss function of the model was optimized. The original loss function of the YOLO v5 model was GIoU, which could not accurately reflect the distance between the real box and the predicted box in some cases. In contrast, the CIoU loss function takes into account multiple factors such as the overlapping area of the box, the scale, direction of the box, and the center point distance, which can more accurately measure the difference between the boxes in wheat leaf detection, further improving the accuracy of wheat leaf detection.

### 2.4 Wheat leaf detection based on Wheat-RYNet model

#### 2.4.1 Technical route

**Figure 7:**
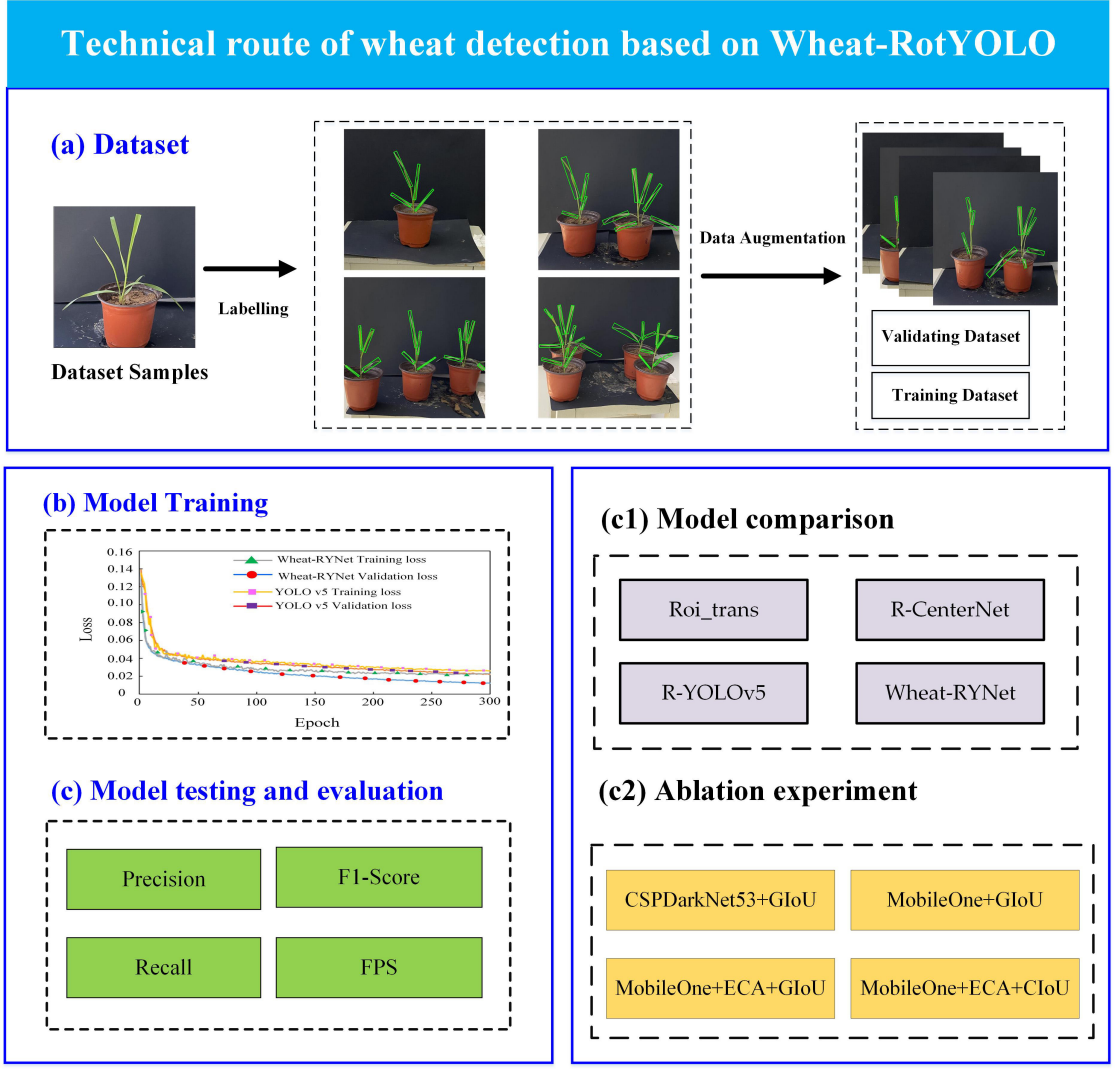
Technical route of wheat leaf detection based on Wheat-RYNet model. **(a)** Dataset, **(b)** Model Training, **(c)** Model testing and evaluation, **(c1)** Model comparison, **(c2)** Ablation experiment.

**Figure 7** shows in detail the high-throughput wheat phenotypic traits extraction process based on the Wheat-RYNet model. The specific steps are as follows:

To begin with, a comprehensive wheat sample dataset was meticulously curated. Each sample within this dataset underwent a rigorous and professionally guided semantic annotation process, ensuring a high - quality data foundation that would serve as the bedrock for subsequent model training endeavors.

In the subsequent phase, the Wheat - RYNet model was employed to undertake the crucial task of wheat seedling target detection. A thorough and systematic comparative analysis was then conducted between the Wheat-RYNet model and several mainstream target detection models, including Roi_trans, R-CenterNet, R_YOLOv5. This analysis focused on evaluating the performance of each model in the specific context of wheat phenotypic feature recognition. The evaluation was carried out from two key dimensions: detection accuracy, which reflects the model’s ability to correctly identify wheat seedlings, and feature extraction capability, which determines how effectively the model can capture the relevant phenotypic traits of the wheat seedlings.

Additionally, ablation experiment was put into practice. This experiment involved the strategic removal or substitution of core components within the models, such as CSPDarkNet53, MobileOne, and ECA. By doing so, it was possible to conduct a quantitative analysis of the impact these components had on both the detection accuracy and generalization ability of the models. This process played a pivotal role in the optimization and iterative refinement of the Wheat - RYNet model’s structure, ensuring that it was as efficient and effective as possible.

Finally, a comprehensive assessment was performed on the Wheat - RYNet model. The evaluation encompassed several critical metrics, namely detection accuracy, the F1 value, the recall rate, and the inference frame rate (FPS), which measures the model’s speed in processing images. This evaluation was essential to guarantee the model’s reliability and effectiveness when deployed in real - world agricultural production scenarios.

#### 2.4.2 Model evaluation metrics

For the wheat leaf detection task, five core evaluation indicators are used to comprehensively and deeply analyze the performance of different models, as shown in Equations 7-11. These five indicators are: average precision (AP), which not only measures the model’s ability to detect wheat leaves at different intersection-over-union (IoU) thresholds, but also provides a more comprehensive performance evaluation through averaging. Recall, which intuitively reflects the model’s ability to identify and mark all wheat leaf samples. Precision, that is, the proportion of real wheat leaves in the samples predicted by the model as wheat leaves, is an important yardstick for evaluating classification accuracy. F1-Score, as a comprehensive indicator that balances precision and recall, is particularly suitable for evaluating possible category imbalances in wheat leaf detection. Finally, frames per second (FPS) directly measures the speed of the model in processing wheat leaf images, and is a key parameter for evaluating the actual application efficiency of the model. The comprehensive consideration of these five indicators ensures our comprehensive and accurate evaluation of the performance of the wheat leaf detection model. Mean average precision (mAP) is the average of the AP values ​​of all categories, which indicates the overall detection performance of the model on all categories.


(7) $$\text{Precision}=\frac{TP}{TP+FP}$$

(8) $$\text{Recall}=\frac{TP}{TP+FN}$$

(9) $$\text{F}1-\text{score}=\frac{2\times \text{Precision}\times \text{Recall}}{\text{Precision}+\text{Recall}}$$

(10) $$mAP=\frac{1}{\left|k\right|}\sum_{i}^{k}AP_{i}$$

(11) $$\;AP=\int_{0}^{1}\text{Precision}(\text{Recall})\text{dR}$$

In the formula, k represents the number of categories of the target to be detected, TP indicates the number of wheat leaves correctly detected and located by the model, FP indicates the number of wheat leaves incorrectly detected by the model, and FN indicates the number of wheat leaves not detected by the model.

#### 2.4.3 Experimental environment

The hardware equipment of the experiment was mainly configured with Intel Core i7–8700 CPU @ 3.20GHz and NVIDIA GeForce RTX 2080 Ti GPU with 16GB video memory. The operating system used was Windows 11 with Python 3.8 and Pytorch 1.12.1 installed. The hyperparameters of network training are as follows: the training process is 300 epochs, each batch contains 8 samples; the learning rate is 0.01, the momentum is 0.937, and SGD is used as the optimization algorithm; at the same time, the weight decay is set to 0.0005, and the size of all images is uniformly adjusted to 640×640.

## 3 Results

### 3.1 Wheat leaf detection results based on Wheat-RYNet

**Figure 8:**
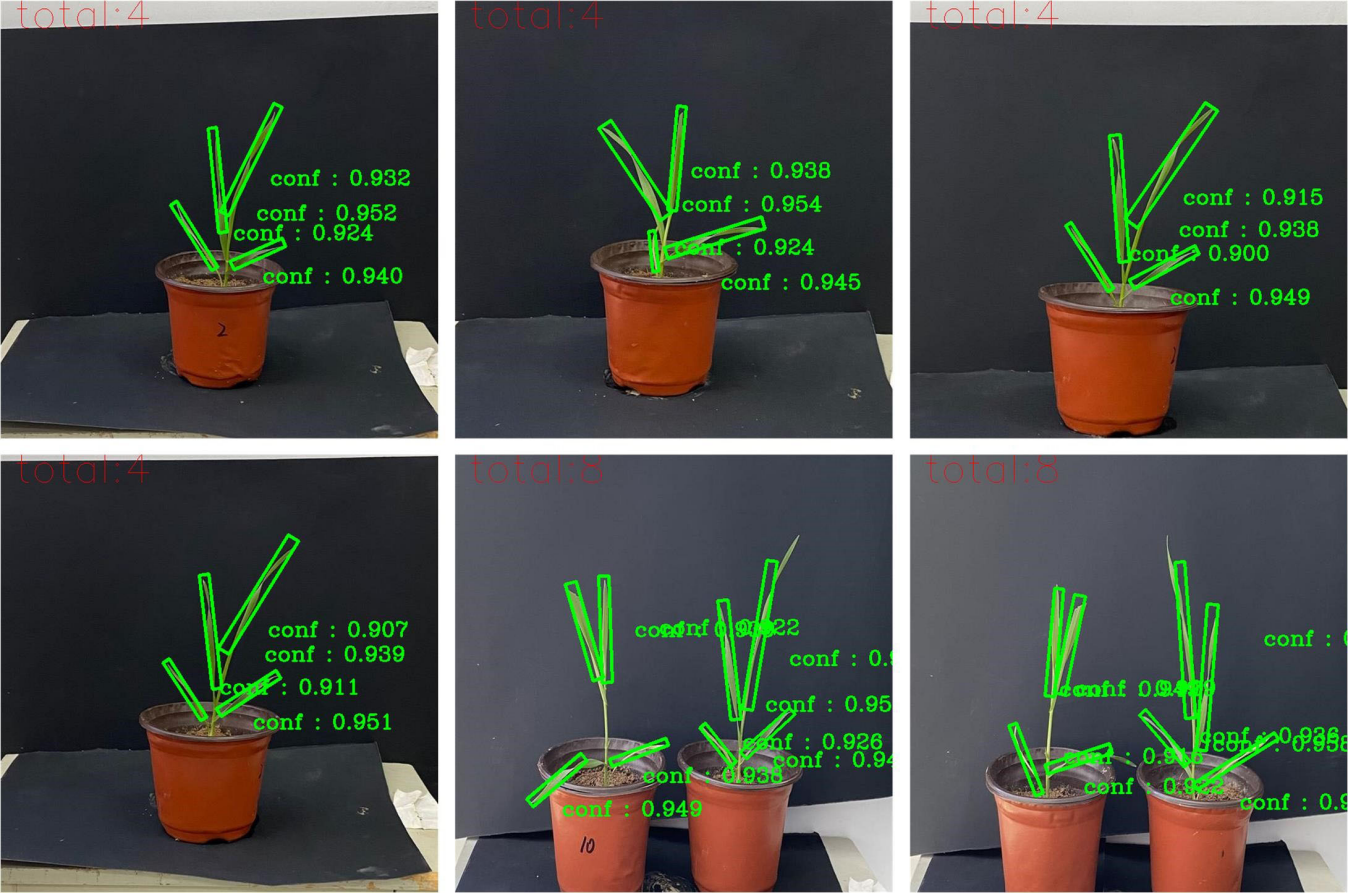
Detection results of the Wheat-RYNet model.

A self-built dataset containing 1220 wheat images was used as the training set of the Wheat-RYNet model to improve the model’s ability to detect wheat leaves. After the training was completed, the optimal weight of the model was used to test the wheat test set containing 122 images to verify its generalization ability. The test results showed that the model performs well in wheat leaf detection. The detection result example is shown in **Figure 8**. We can intuitively see from **Figure 8** that the model can accurately locate and select the wheat leaves in the image. Even small leaves can be effectively identified, which fully demonstrates the effectiveness and reliability of the model in practical applications.

### 3.2 Extracting phenotypic traits based on image detection

**Figure 9:**
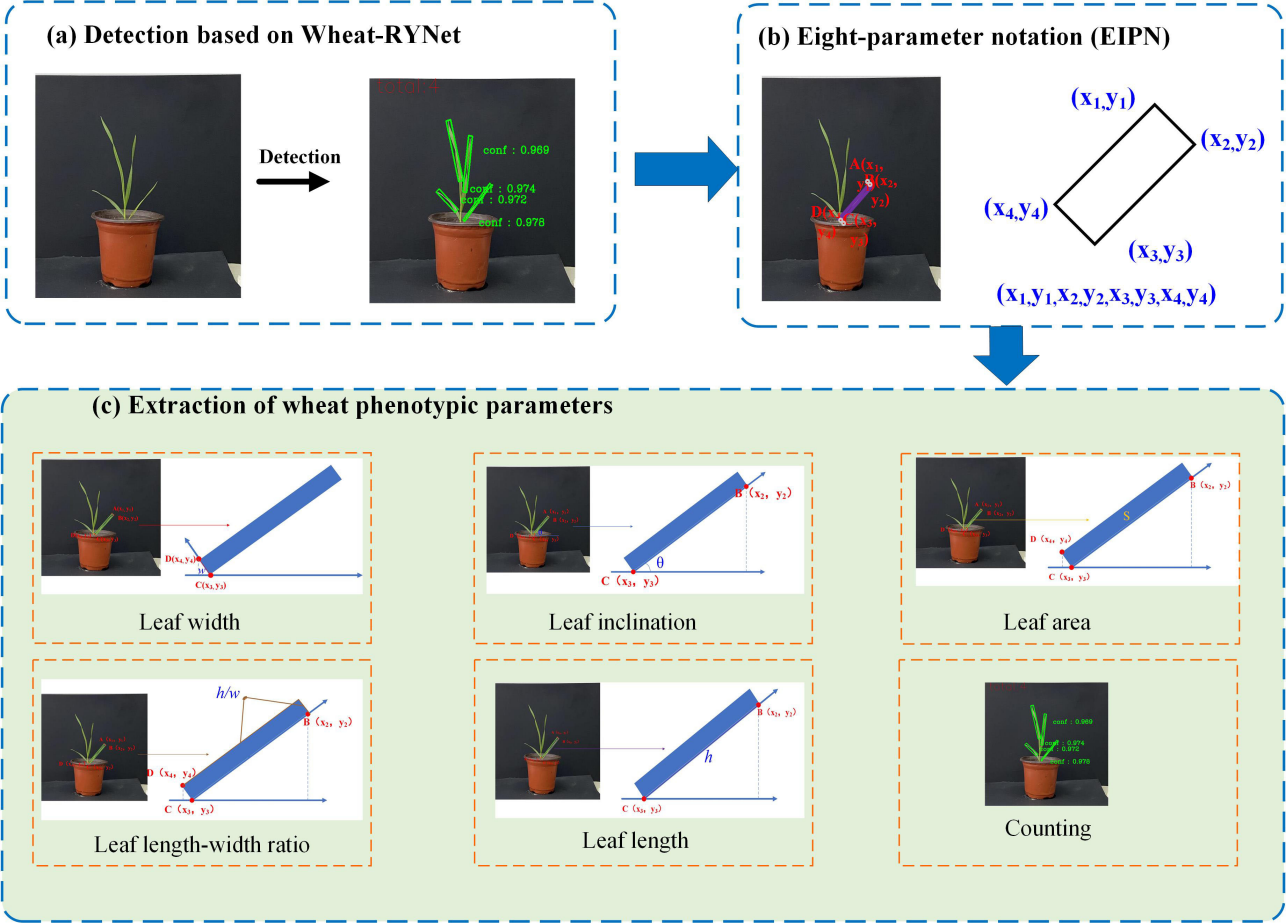
Flow chart of wheat plant phenotypic traits extraction: **(a)** Detection based on Wheat-RYNet; **(b)** Eight-parameter notation (EIPN); **(c)** Extraction of wheat phenotypic trait.

To quickly and accurately extract the diverse phenotypic traits of wheat leaves, we innovatively integrated the Wheat-RYNet rotation target detection model to achieve fine recognition and precise positioning of each leaf in the wheat leaf image. The specific process is shown in **Figure 9**.

Firstly, Wheat-RYNet is used for detection to obtain the detection confidence of the crop. Then, the posture of the crop is described by the eight-parameter notation method (EIPN) to obtain the coordinates of each point. Finally, based on this coordinate information, the phenotypic traits of the crop are extracted, such as leaf width, leaf inclination, leaf area, leaf length, leaf length ratio, and leaf number. The model accurately detected wheat leaves and calculated the relevant parameters according to the formula in **Table 1**.

**Table 1:** Formula for extracting phenotypic trait.

Wheat plant phenotypic trait	Calculation formula
*θ*	$\arctan(\frac{\left|y_{2}-y_{3}\right|}{\left|x_{2}-x_{3}\right|})$
*h*	$\sqrt{{\left(x_{2}-x_{3}\right)}^{2}+{\left(y_{2}-y_{3}\right)}^{2}}$
*w*	$\sqrt{{\left(x_{4}-x_{3}\right)}^{2}+{\left(y_{4}-y_{3}\right)}^{2}}$
h/w	$\sqrt{{\left(x_{2}-x_{3}\right)}^{2}+{\left(y_{2}-y_{3}\right)}^{2}}/\sqrt{{\left(x_{4}-x_{3}\right)}^{2}+{\left(y_{4}-y_{3}\right)}^{2}}$
*S*	$\sqrt{{\left(x_{2}-x_{3}\right)}^{2}+{\left(y_{2}-y_{3}\right)}^{2}}\times \sqrt{{\left(x_{4}-x_{3}\right)}^{2}+{\left(y_{4}-y_{3}\right)}^{2}}$

Among them, *θ* is the leaf inclination angle, *h* is the leaf length, *w* is the leaf width, h/w is the leaf length-to-width ratio, *S* is the wheat leaf area, $\left(x_{1},y_{1}),(x_{2},y_{2}),(x_{3},y_{3}),(x_{4},y_{4}\right)$ are the coordinates of the four vertices of the leaf detection box.

**Figure 10:**
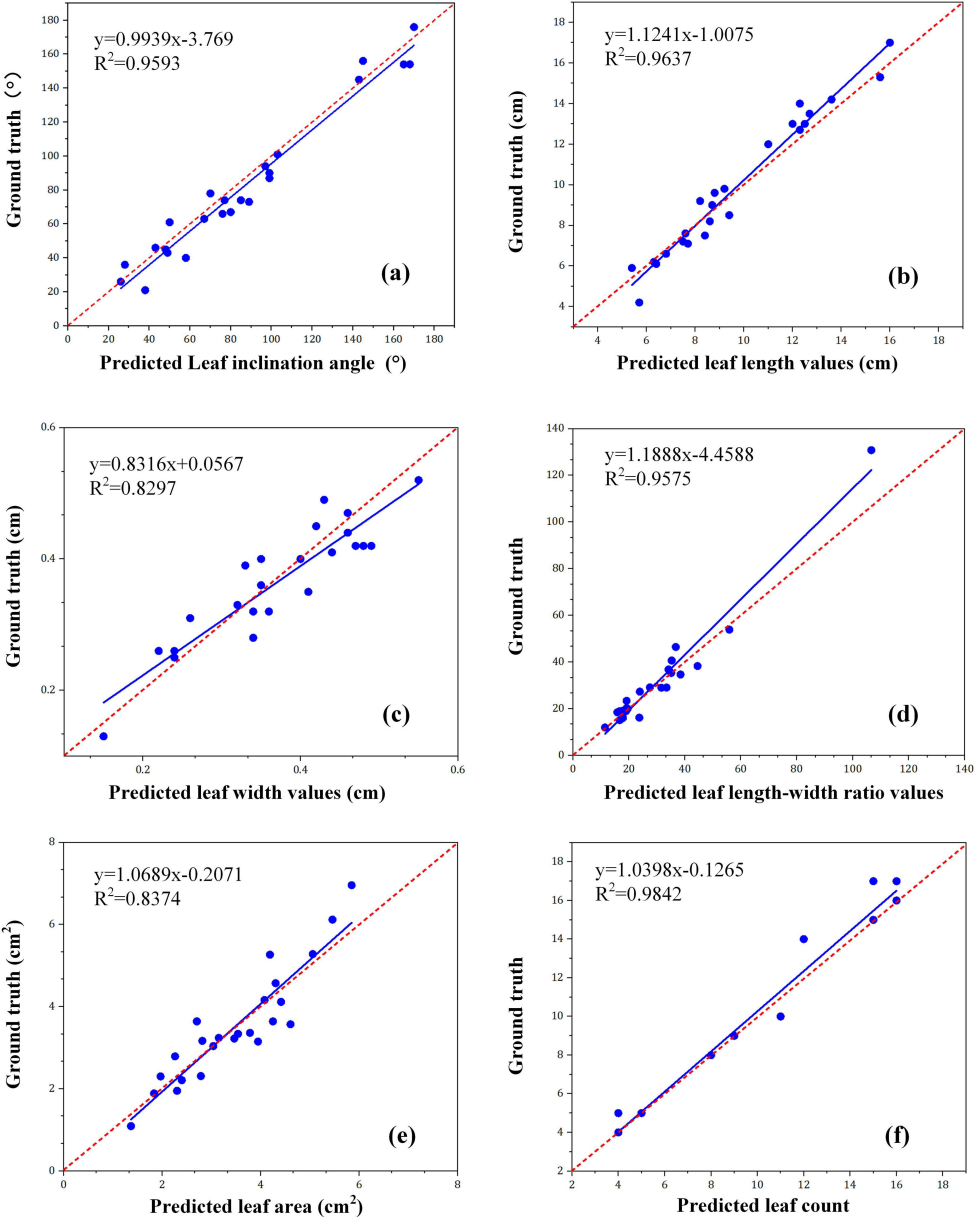
The test results of wheat phenotypic traits, including **(a)** leaf inclination angle; **(b)** leaf length; **(c)** leaf width; **(d)** leaf length-to-width ratio; **(e)** leaf area; and **(f)** leaf count, where R2 is the absolute coefficient.

To evaluate the accuracy of the Wheat-RYNet model in wheat phenotypic analysis, 10 wheat plants were selected as test samples and tested using this model. The number of detection bounding boxes was counted as the total number of predicted wheat leaves. Subsequently, 24 rotated detection bounding boxes of wheat leaves were randomly selected. The vertex coordinates of these rotated detection bounding boxes were calculated using formulas and normalized to obtain the model calculation results of leaf inclination angle, leaf length, leaf width, length-width ratio, and leaf area. Finally, these data were compared and evaluated with the ground truth values. The specific evaluation results are shown in **Figure 10**.

As can be seen from **Figure 10**, the R^2^ value is 0.9593, 0.9637, 0.8297, 0.9575, 0.8374, and 0.9842, respectively, indicating that the Wheat - RYNet rotation target detection model can well detect wheat plant phenotypic traits with high accuracy. From **Figure 10**, we also found that the prediction accuracy of leaf width is slightly lower, probably because the image obtained is a side view. This perspective makes it difficult to accurately capture the true width of the leaf, the fact which in turn affects the estimation accuracy of the leaf area. To improve this situation, although obtaining a top view may be an effective way because it can provide more accurate leaf width information, doing so may also bring challenges to the estimation of leaf length. RGB cameras have been widely used in many application fields due to their low cost, convenient operation, reliable imaging quality and excellent environmental adaptability (Wu et al., 2022). For example, the accurate extraction of rice plant height information benefits from the combination of the server and the RGB camera (Sritarapipat et al., 2014). In recent years, deep - learning - based technologies have made significant progress in using RGB images for wheat phenotyping and have yielded a series of results. For example, Mask R - CNN is used to accurately extract wheat information from RGB images (Machefer et al., 2020). Therefore, in the future, it is necessary to consider various factors and find ways to improve the accuracy of leaf width estimation while maintaining the accuracy of leaf length estimation.

## 4 Discussion

### 4.1 Detection results based on different rotation box models

Most deep learning-based object detection (Girshick et al., 2014; He et al., 2015; Ren et al., 2015) implements target detection based on horizontal bounding box (HBB). However, rotated bounding box (RBB) (Yang et al., 2019a) greatly improves the detection efficiency of objects by introducing angle parameters. To comprehensively and fairly evaluate the performance advantages of the Wheat-RYNet model in the wheat leaf detection task, a wheat leaf detection comparison experiment on four detection models was designed, including the rotation target detection model Roi_trans (RoI Transformer), the key point-based R-CenterNet, the R-YOLOv5, and the Wheat-RYNet proposed in this study. All models were trained and tested in a unified experimental environment using the same wheat leaf image set to eliminate the influence of external factors on the evaluation results. The experimental results are summarized in **Table 2**, which shows in detail the specific performance of each model in key indicators such as mAP (mean average precision), Recall, Precision, and F1-Score, which intuitively reflects the excellent performance of the Wheat-RYNet model in the wheat leaf detection task.

**Table 2:** Performance comparison of different rotating target detection models.

Model	mAP	Recall	Precision	F1-score
Roi_trans	0.738	0.783	0.808	0.795
R-CenterNet	0.764	0.795	0.832	0.813
R_YOLOv5	0.805	0.825	0.847	0.836
Wheat-RYNet	**0.833**	**0.851**	**0.863**	**0.857**

The bold part indicates the best effect among several models.

According to the detailed data analysis in **Table 2**, the Roi_trans rotation object detection model performed poorly in the wheat leaf detection task, with its F1-Score (0.795) and AP (0.738) at the lowest level, showing lower detection performance compared with the other three models. In contrast, the R-CenterNet model has made progress in wheat leaf detection, with an F1-Score of 0.813 and an AP of 0.764, which are 1.8% and 2.6% higher than the Roi_trans model, respectively. Furthermore, the YOLOv5 rotation object detection model surpassed the R-CenterNet in detection effect, with an F1-Score of 0.836 and an AP of 0.805, achieving a significant increase of 2.3% and 4.1% over the R-CenterNet model. The Wheat-RYNet rotation target detection model proposed in this study, while maintaining the high efficiency of R- YOLOv5, has achieved a further leap in performance by introducing optimization methods such as MobileOne and ECA. Specifically, the model has improved 4.4% (reaching 0.857) compared with R-YOLOv5 and 6.6% compared with R-CenterNet in F1-Score; it has improved 2.8% (reaching 0.833) compared with R-YOLOv5 and 8.5% compared with R-CenterNet in AP. In summary, the Wheat-RYNet rotation target detection model stands out among all compared models with its excellent F1-Score and AP values, fully demonstrating its applicability and efficiency for wheat leaf detection tasks.

**Figure 11:**
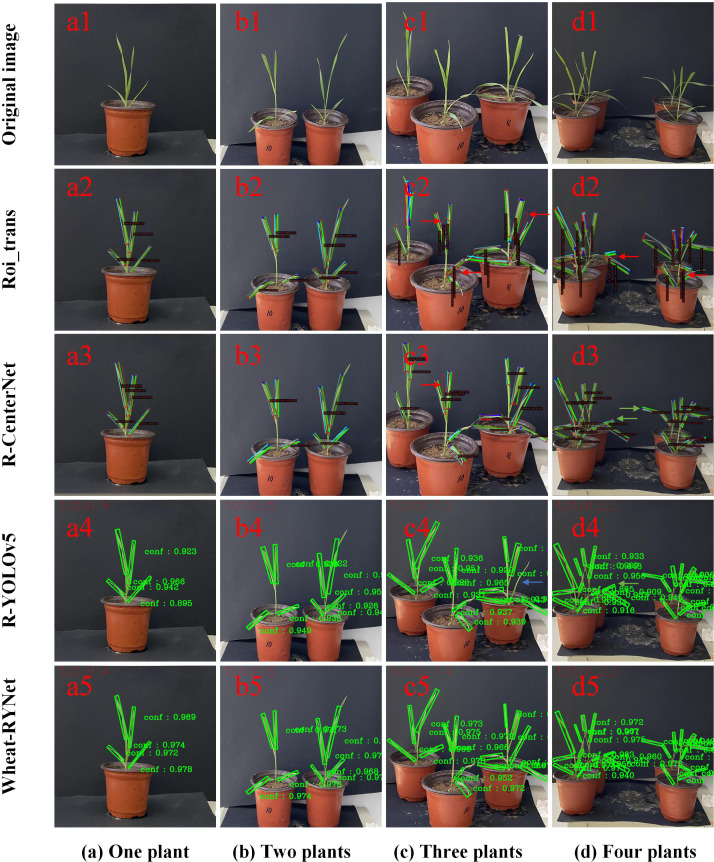
Detection results of different numbers of wheat leaves. **(a)** One plant, **(b)** Two plants, **(c)** Three plants, **(d)** Four plants.

To intuitively and specifically demonstrate the actual effect of each model in the wheat leaf detection task, we randomly selected several wheat pictures from the dataset and presented the detection results of different models on these images, as shown in **Figure 11**. The models are compared in terms of recognition accuracy, bounding box positioning accuracy, and the ability to handle complex backgrounds.

**Figure 11** intuitively shows the significant improvement in target detection confidence of the Wheat-RYNet rotating target detection model compared with Roi_trans, R-CenterNet and YOLOv5. As the number of wheat plants increases, the occlusion between leaves intensifies, and the difficulty of rotating target detection also increases, which often leads to problems such as false detection, re-detection and missed detection. Specifically, in **Figure 11**, the false detection of the Roi_trans model (as shown by the red arrow in c2) reveals its limitations in complex scenes. The R-CenterNet and YOLOv5 models face the challenge of re-detection (green arrows in d3, d4, and d5), indicating that these models still need to be optimized when dealing with dense and occluded targets. In addition, the YOLOv5 model also has missed detection (blue arrow in c4), further highlighting the importance of improving detection accuracy.

However, after the introduction of the Wheat-RYNet model, the model can still maintain a high detection performance even when the number of wheat leaf increases and the leaves are severely occluded. The confidence of the detected target is generally above 0.9, which is mainly due to the introduction of the ECA attention mechanism. The ECA mechanism effectively emphasizes the key feature channels related to wheat detection, while suppressing the interference of irrelevant background information, thereby improving the discrimination ability of the model. In addition, the adoption of the CIoU loss function further optimizes the regression process of the bounding box and ensures the accuracy and stability of the detection box. In summary, the Wheat-RYNet model significantly improves the wheat leaf detection ability in complex scenes by combining the ECA attention mechanism and the CIoU loss function, effectively reducing the occurrence of false detection, re-detection and missed detection, and providing strong support for the accurate extraction of wheat leaf phenotypic traits.

### 4.2 Ablation experiment

Studies have shown that YOLOv5 combines different backbone networks, such as CSPNet (Wang et al., 2020a), EfficientNet (Tan and Le, 2019), and different Neck networks, such as PANet (Liu et al., 2018), FPN (Lin et al., 2017), etc., to significantly improve its capabilities in image feature extraction and object detection. In this study, to enhance the detection accuracy and speed of YOLOv5, the Wheat-RYNet model is proposed, which uses the MobileOne network as the backbone and introduces the attention mechanism.

To fully and deeply verify the performance advantage of the Wheat-RYNet model in the target rotation detection task, we designed a set of ablation experiments. The experiment is divided into 4 groups, C1-C4, for training: C1 is the YOLOv5 model; C2 is the feature backbone network of the YOLOv5 model replaced with the Mobileone module; C3 is the ECA attention mechanism embedded in the neck network of the YOLOv5 model; C4 is the ECA attention mechanism embedded in the neck network of the YOLOv5 model, and the CIoU loss function is used. The experiment focuses on three modules: the MobileOne network, the ECA attention mechanism, and the CIoU loss function. The ablation experiment is carried out using the same wheat leaf dataset under the same experimental environment to clarify the specific contribution of each module to the overall performance of the model. The performance comparison results of the ablation experiment are shown in **Table 3**.

**Table 3:** Performance comparison of Wheat-RYNet ablation experiment.

Class	CSPDarkNet	Mobileone	ECA	GIoU	CIoU	mAP	F1-score	FPS(fs^-1^)
C1	✓	×	×	✓	×	0.805	0.836	37
C2	×	✓	×	✓	×	0.799	0.830	52
C3	×	✓	✓	✓	×	0.828	0.846	50.2
C4	×	✓	✓	×	✓	0.833	0.857	50.2

From the experimental results in **Table 3**, it can be seen that the YOLOv5 model (classification: C1) shows good performance in the wheat leaf detection task, with mAP and F1-score reaching 80.5% and 83.6%. To further improve the model in real-time processing, the MobileOne module is introduced into the backbone network of the model. As a result, the mAP and F1-score dropped by 0.75% and 0.72% respectively, but the accuracy loss was controlled within 1%, maintaining a high detection level. However, the introduction of the MobileOne module significantly improved the computational efficiency of the model, with the FPS increased by 40.5%, achieving a leap in running speed.

Furthermore, the ablation experiment results show that the model performance has been significantly improved by combining the MobileOne backbone network, with mAP and F1-score increased by 3.5% and 1.9% respectively. The experiment shows that the MobileOne module has a significant effect in optimizing computing resources and accelerating the computing process. In addition, the ECA attention module is introduced to model the long-range dependencies between channels through learnable one-dimensional convolution operations (Khotimah et al., 2022), thereby enhancing the feature expression of important channels and suppressing the influence of unimportant channels.

In terms of loss function, CIoU is selected to replace the traditional GIoU. CIoU comprehensively considers the overlap, center point distance and aspect ratio difference, and is more suitable for processing the variable shape, size and field distribution of wheat leaves. Experiments have shown that after using CIoU as the loss function, the mAP and F1-score of the model are improved by 3.4% and 2.5% respectively, significantly reducing false detection and missed detection, and improving detection accuracy and efficiency.

### 4.3 Limitations and prospects

#### 4.3.1 Limitations

Although the “CropPhenoX” system leveraging YOLOv5 has achieved significant progress in wheat leaf detection and phenotypic traits extraction, substantial opportunities for enhancement remain in both system performance and functionality. Currently, the system’s exclusive reliance on RGB image data limits its capacity to integrate multimodal information, impeding comprehensive analysis of crop physiological states. Additionally, the absence of multi-user collaborative operation and cross-platform data sharing capabilities within the software platform restricts data exchange efficiency and collaborative productivity.

Specifically, the choice of YOLOv5 over YOLOv8 and subsequent versions for wheat leaf detection within the “CropPhenoX” system was meticulously informed by three key factors. Firstly, the YOLOv5 community is rich in resources, with a large number of practical experiences, code examples and pre-trained models for agricultural image analysis, which allows the research team to quickly learn from them to solve problems. However, newer versions such as YOLOv8 have fewer mature application cases in the agricultural field and insufficient reference. Secondly, YOLOv5 has a variety of lightweight versions that can adapt to hardware with different computing capabilities at low computing costs and fast reasoning speeds, ensuring that the system runs efficiently in high-throughput detection scenarios (Xu et al., 2023). In contrast, YOLOv8 and above versions have higher requirements for hardware resources, which may affect performance when hardware is limited (Yan et al., 2024). Finally, the YOLOv5 code structure is clear, which is conducive to the modification and expansion of the “CropPhenoX” system to meet the special needs of wheat leaf detection.

In fact, during the application process, the stability of technology is far more highly valued than new functions. Especially in scenarios where real - time performance is prioritized and hardware resources are limited, a lightweight version should be chosen (Liu et al., 2024). Therefore, the lightweight YOLOv5 model is selected in this article. Thus, optimal system performance in specific scenarios can only be ensured by comprehensively considering the technical ecology, hardware adaptability, and development flexibility. Of course, with the deepening of agricultural phenotyping research and the iterative upgrading of hardware technology, the dynamic optimization and cross - version integration of the model also needs to be continuously explored in the future, so that the detection accuracy and application universality of the system can be further enhanced.

#### 4.3.2 Prospects

To further expand the depth and breadth of the research, the following four dimensions can be explored in the future:

Firstly, to enhance model interpretability, we will systematically analyze the feature extraction logic and decision-making mechanism of the Wheat-RYNet model using advanced visualization technologies such as Grad-CAM and SHAP. We will intuitively present the model’s recognition patterns for phenotypic features like wheat leaf morphology and texture, transforming the “black-box” process of deep learning into interpretable visual results to provide a scientific basis for breeding decisions.

Secondly, to expand cross-crop application boundaries, we will optimize the model’s feature extraction module for major crops such as rice and maize. Combining domain adaptation algorithms and few-shot learning techniques, we will transfer the rotated box detection strategy to phenotypic analysis scenarios of different crops, constructing a universal crop phenotyping framework to further broaden the technical application scope.

Thirdly, to improve phenotyping measurement accuracy, we will adopt a fusion scheme of 3D point cloud and multi-view imaging. By using top-view imaging to reduce the interference of angle deviations on leaf width measurement, and fusing 3D point cloud data to reconstruct the three-dimensional morphology of leaves, we aim to enhance the extraction accuracy of phenotypic traits such as leaf width from a stereoscopic perspective, thereby improving the accuracy and robustness of crop phenotyping analysis.

## 5 Conclusion

In this study, a software-hardware collaborative automated phenotypic extraction system for wheat was successfully constructed. High-throughput acquisition of wheat seedling images was achieved through the CropPhenoX hardware platform. The Wheat-RYNet detection model and the automated extraction system enabled accurate identification of phenotypic parameters, effectively overcoming the efficiency and accuracy bottlenecks of traditional methods. Experimental results demonstrate that the system outperforms existing technologies in terms of phenotypic parameter extraction, target detection, and other related indicators, providing reliable technical support for wheat genetic breeding and field management. Nevertheless, limitations exist in multimodal data fusion and multi-user collaboration. In future research, efforts will be made to enhance the hardware’s environmental adaptability, improve algorithm generalization capabilities, and expand software functionality, thereby advancing wheat phenotyping technology towards greater intelligence and standardization.

## Data Availability

The raw data supporting the conclusions of this article will be made available by the authors, without undue reservation.
